# A phantom decoy shifts wild forager decisions in a natural environment

**DOI:** 10.1093/beheco/arag059

**Published:** 2026-05-30

**Authors:** Gabrielle R Jarvis, Peter B Banks, Tanya Latty, Malcolm Possell, Cristian Gabriel Orlando, Clare McArthur

**Affiliations:** School of Life and Environmental Sciences, The University of Sydney, Heydon-Laurence Building A08, Science Rd., Camperdown, Sydney, NSW 2050, Australia; School of Life and Environmental Sciences, The University of Sydney, Heydon-Laurence Building A08, Science Rd., Camperdown, Sydney, NSW 2050, Australia; School of Life and Environmental Sciences, The University of Sydney, Heydon-Laurence Building A08, Science Rd., Camperdown, Sydney, NSW 2050, Australia; School of Life and Environmental Sciences, The University of Sydney, Heydon-Laurence Building A08, Science Rd., Camperdown, Sydney, NSW 2050, Australia; School of Life and Environmental Sciences, The University of Sydney, Heydon-Laurence Building A08, Science Rd., Camperdown, Sydney, NSW 2050, Australia; School of Life and Environmental Sciences, The University of Sydney, Heydon-Laurence Building A08, Science Rd., Camperdown, Sydney, NSW 2050, Australia

**Keywords:** decision-making, ecological rationality, foraging, heuristics, swamp wallaby, *Wallabia bicolor*

## Abstract

Seemingly irrelevant options, such as inferior or unavailable “decoys”, can shift decisions people and captive animals make in simplified environments. But do decoys influence animals in messy, complex, natural environments? Here, we explored this question in a foraging context, a process involving countless daily decisions that ultimately underpins animal fitness. We tested whether a “phantom decoy” (inaccessible but preferred food) influenced food choice of free-ranging swamp wallabies across 3 scenarios; *Binary—*2 available, equally preferred foods differing nutritionally; *Phantom—*2 available foods plus an inaccessible phantom decoy nutritionally similar to one; *Trinary*—all 3 foods available. We analysed data using a new approach that overcame cross-level bias, when individual choices shift in opposite directions leading to no overall shifts. We found that the phantom decoy made wallabies more likely to switch their choice away from the nutritionally similar food—a net reactance effect. Unexpectedly, when all foods were available, wallaby choices appeared random and haphazard, suggesting cognitive overload. These findings demonstrate that decoys can shape ecologically relevant foraging decisions, so their influence is not simply an inadvertent byproduct of stylized simplistic settings. As such, traditional foraging models may better predict real-world foraging decisions by incorporating decoy effects.

## Teaser

We found that wild wallaby food choices were altered by the presence of “irrelevant” options that is, an inaccessible food item. This undermines the assumption that animals always forage optimally, consistently choosing the best available food. We also found that food choice often changed again when choice-set size increased. These findings highlight the power of the choice environment in shaping real-world animal decisions.

## Introduction

Life is defined by decisions, a constant chain of choices including what to eat, who to mate with, where to sleep. Good decisions ultimately lead to enhanced fitness. If decisions were based solely on the absolute value of options, we would simply choose the option with the highest value. Inferior or unavailable options—“decoys”—would have no influence on our choice among options of higher value (Wedell 1991; Pratkanis and Farquhar 1992).

In ecology, classic foraging theories, such as Optimal Foraging Theory (OFT [Charnov 1976; Pyke 1984]) and Bayesian Updating foraging models (McNamara et al. 2006), predict animals choose food with the highest net (“absolute”) value across multiple dimensions—nutrition content, predation risk, time investment and other costs and benefits associated with obtaining them—relative to other foods. The assumption of this “absolute evaluation system” is that animals have, or obtain, precise knowledge of options in the landscape when making foraging decisions. Yet, this may not be possible in spatiotemporally heterogenous, noisy environments with unlimited sources of information (Fawcett et al. 2014). Animals may, instead, consider additional or “irrelevant” information (such as decoys) when making food choices to help process information and choose among options (Marsh 2002; Hutchinson and Gigerenzer 2005). If decoys consistently influence foraging decisions of wild animals in their natural environment, then foraging theory and predictions will be improved by incorporating their effects.

In humans, seemingly irrelevant decoys shift decisions across many domains; from choosing what we buy (Doyle et al. 1999; Pettibone and Wedell 2007; Frederick et al. 2014; Wu and Cosguner 2020; Marini et al. 2025) to how we evaluate risks (Cheng et al. 2012; Mohr et al. 2017; Marini and Paglieri 2019) or make perceptual (Trueblood et al. 2013; Trueblood and Pettibone 2017; Spektor et al. 2018; Liao et al. 2021; Parrish et al. 2024), political (O’Curry and Pitts 1995; Herne 1997), legal (Kelman et al. 1996), and medical (Schwartz and Chapman 1999; Stoffel et al. 2023) decisions.

A diverse range of other taxa also respond to decoys. Decoys affect food choice by slime molds (Latty and Beekman 2011; Yin et al. 2025), bees (Shafir et al. 2002; Tan et al. 2015; Hemingway et al. 2024), birds (Bateson 2002; Bateson et al. 2002; Morgan et al. 2012), and mammals (Scarpi 2011; Hemingway et al. 2021; Jackson and Roberts 2021; Marini et al. 2024); mate choice by fish (Royle et al. 2008; Locatello et al. 2015) and frogs (Lea and Ryan 2015); and nest selection by ants (Sasaki and Pratt 2011). The presence of decoy effects across taxa suggests an evolutionary underpinning for this response and strongly indicates responses to decoys may be adaptive (Hutchinson and Gigerenzer 2005). This selected function may stem from decoys prompting the use of simple cognitive shortcuts or rules of thumb so an animal can make a fast, frugal, and generally “good enough” decision (Marsh 2002; Gigerenzer and Gaissmaier 2011). These cognitive shortcuts may be particularly beneficial when animals have imperfect knowledge about options in the environment or when under marked time pressure (Fawcett et al. 2014).

However, almost all evidence for decoys influencing decision-making in nonhumans comes from captive animals in carefully controlled, lab-based settings. Such settings are highly simplified and bear little resemblance to the complex structure of the natural world. In the wild, free-ranging animals must navigate noisy, messy, fluctuating environments. They are faced with choosing amongst multidimensional options that may also vary in space and time (Fawcett et al. 2014). This raises an important question; do decoys affect decisions about complex items in these more complicated, ecologically relevant scenarios?

The few studies that have investigated the influence of decoys in the wild, report effects consistent with many lab-based outcomes. Using simplified artificial nectar that varied in just 2 clear dimensions (eg, volume and concentration of sucrose) inferior decoys altered foraging choices of free-ranging gray jays (*Perisoreus canadensis*) (Shafir et al. 2002) and rufous hummingbirds (*Selasphorus rufus*) (Bateson et al. 2003; Morgan et al. 2014). In natural open forest, a phantom decoy (present, superior in nutrition, but inaccessible) influenced information-gathering behavior of free-ranging swamp wallabies (*Wallabia bicolor*) when offered complex artificial foods (Orlando et al. 2023). While these studies have begun to investigate context-dependent decisions in wild animals, the impact of decoys on choice in the real-world remains largely unexplored.

Until we understand if and when decoys matter to free-ranging animals when deciding among multidimensional options, it is unclear whether and how cognitive theories of decision-making need be integrated into ecology and, specifically, foraging ecology. Foraging is a critical yet time-consuming behavior involving countless decisions daily. Foraging choices affect an individual's nutritional and energetic state while imposing missed opportunity costs for alternate activities (Brown and Kotler 2004). Foraging decisions, particularly by herbivores, also have broader ecological consequences such as influencing plant community structure and distribution through trophic cascades (McArthur et al. 2014; Ripple et al. 2015). As such, understanding what information animals consider, and how they evaluate options when making food choices (Stephens 2008) should help predict the decisions they make but also the broader implications of these decisions.

Here, we tested the impact of a phantom decoy on foraging decisions of a free-ranging mammalian herbivore, the swamp wallaby, when offered complex multidimensional foods in a backdrop of native open forest as in Orlando et al. (2023). Foraging by swamp wallabies in Australia (Foster et al. 2015; Morgan 2021), and by ecologically similar herbivores elsewhere such as some deer (Rooney 2001; Cote et al. 2004) and moose (McInnes et al. 1992) in North America, and elephants in Africa (Guldemond and Van Aarde 2008; Daskin et al. 2016), can shape the structure and composition of plant communities. Therefore, it is critical to understand the factors influencing the foraging decisions of these species.

**Figure 1:**
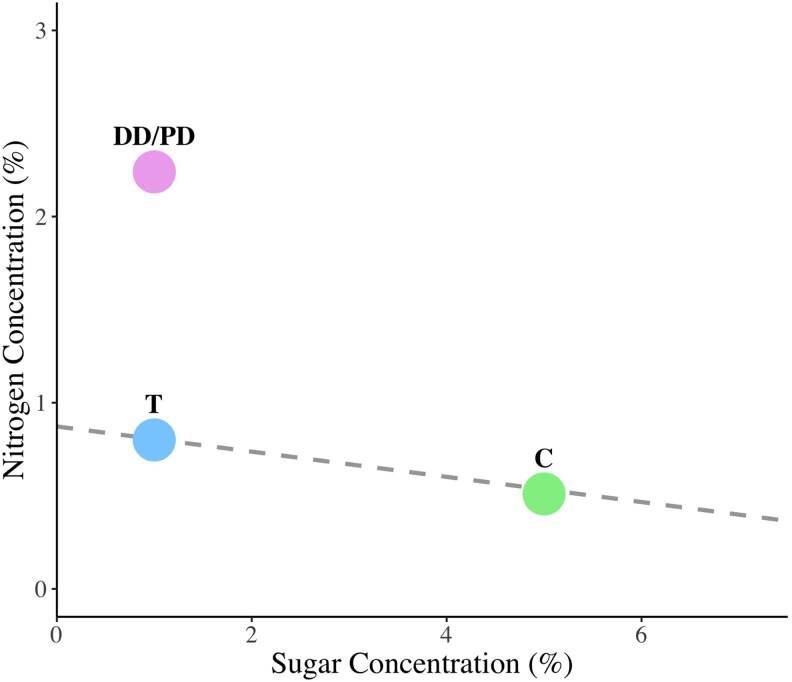
Location of artificial foods in nutritional space, as defined on 2 dimensions: sugar and nitrogen (% concentration in respective dry foods). Dominating decoy (DD when available)/phantom decoy (PD when unavailable), target (T) and competitor (C). The dotted line represents food locations of equivalent nutrient value—not the relative value of nutrients is nonlinear.

Specifically, we asked whether and how the presence of a phantom decoy influences choice outcomes of wallabies. We used a 3 phase design. In *Phase 1* we ran a series of binary preference trials to find appropriate foods consistent with typical decoy studies where options are defined on 2 dimensions ([Pettibone and Wedell 2007; Trueblood and Pettibone 2017], Fig. 1); the “target” and “competitor” are equally preferred; the dominating decoy is preferred to both—superior in value to the target on its best dimension and equal in value on the second dimension. The dominating decoy is classed as a “phantom decoy” when it is made unavailable for choice. In *Phase 2,* we ran the main experiment. We compared 3 treatments: *Binary*—available target and competitor, *Phantom*—available target and competitor, plus phantom decoy, and *Trinary*—available target and competitor, plus available dominating decoy.

We included the Trinary treatment to account for experimental design details (see methods), as we expected wallabies to predominantly choose the high value available dominating decoy food. However, the increase in choice set size by 50% may have exceeded the information processing limits of wallabies, incidentally enabling a test for potential cognitive overload (Chernev et al. 2015). This phenomenon is highly documented in human literature and preliminary research on nonhuman species has found such effects in ant nest selection (Sasaki and Pratt 2012), mosquito oviposition (Sharma and Isvaran 2023), bumblebee foraging efficiency (Austin et al. 2019), and cricket mate choice (Tanner and Simmons 2022).

**Figure 2:**
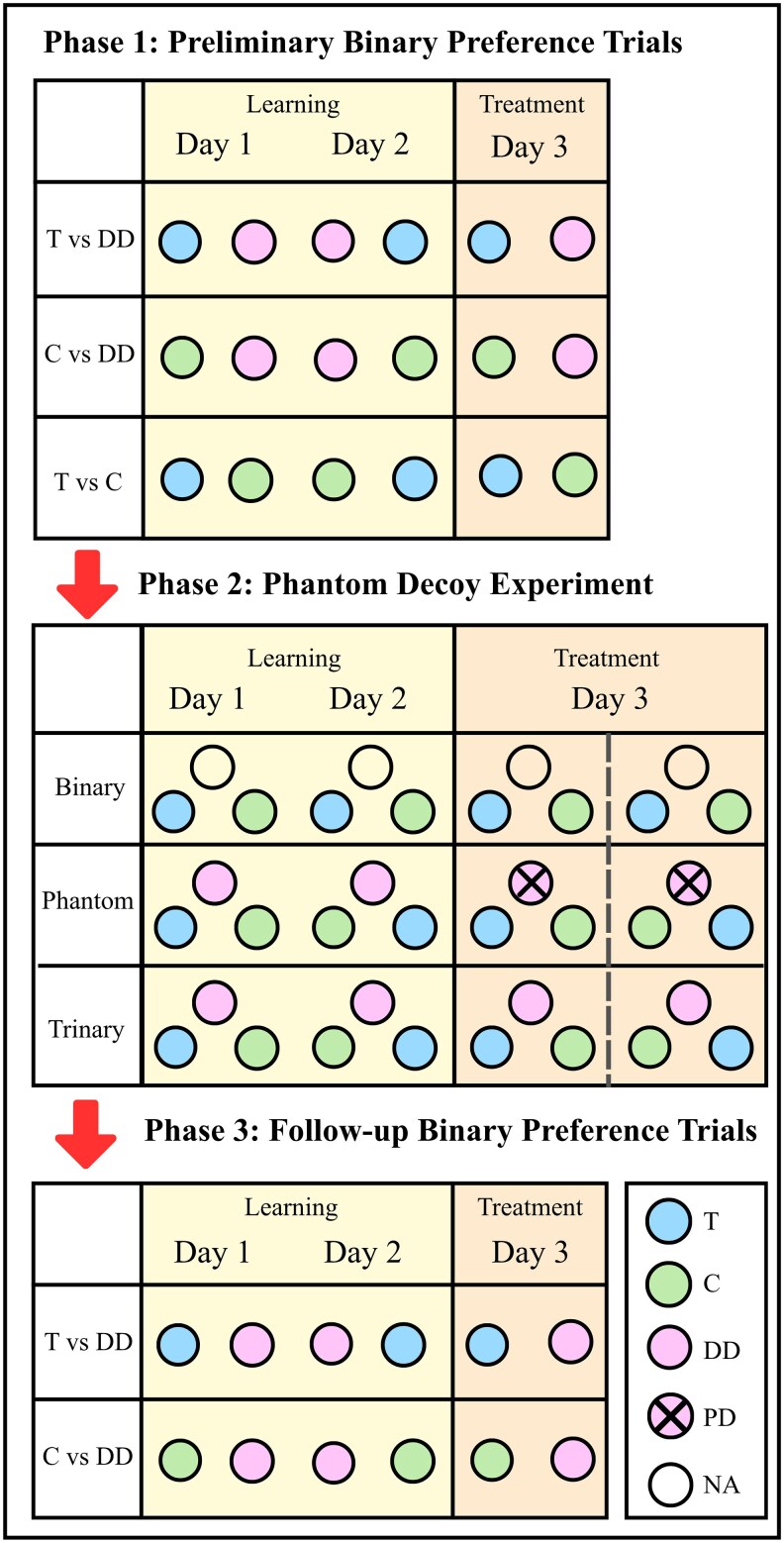
Schematic diagram of experimental study design. Dots represent food items with options being presented in either a binary (Phase 1/Phase 3) or trinary (Phase 2) design per day. Across the 3 phases, the Learning period was on Days 1 and 2, followed by the Treatment Period on Day 3, the valid treatment day. Phase 1 and Phase 3 depicts the format for the binary preference trials and Phase 2 depicts the format for each treatment.

*In Phase 3,* we ran 2 follow-up binary preference trials, comparing the dominating decoy against target and competitor independently, to confirm the dominating decoy was still the most preferred food in a binary setting (Fig. 2).

For the main experiment in *Phase 2*, between the Binary to Phantom treatments we expected a shift in overall choice between target and competitor foods. It is unclear which direction the shift would be as empirical studies have found both a net increase in choice for the target (similarity-substitution effect) and competitor (reactance effect; [Frederick et al. 2014; Spektor et al. 2021]). Between the Binary to Trinary treatments, we predicted a shift in choice towards the available dominating decoy food, since it was highly preferred and of higher nutritional value than the target and competitor foods.

In *Phase 2*, to quantify overall shifts in food choice we first investigated responses to different treatments at the group level (here, wallabies), as is common for phantom decoy studies. However, this approach can lead to errors in inference from cross-level bias, in which individual shifts in choice can be hidden by group-level stability (Robinson 2011). We overcame this problem by implementing a second, new approach of examining individual shifts in food choice, in either direction, between treatment pairs.

## Methods

### Study site and Species

We ran the study in Ku-Ring-Gai Chase National Park, in native open forest and woodland dominated by *Eucalyptus haemastoma*, *Corymbia gummifera*, along with other *Eucalyptus, Banksia*, *Allocasuarina* and, *Casuarina* species and a complex shrub layer. We used free-ranging swamp wallabies, a midsize (13–17 kg) native Australian herbivore as our model species for this study.

### Food preparation

We created complex, multidimensional artificial foods by combining rabbit pellets (“Peckish Guinea Pig and Rabbit” 2.3% N) and oaten hay (“Pete's” 0.44% N), both ground (<2 mm), with raw sugar, and mixed with water to make a paste (Table S1). The foods differed across 2 defined nutritional dimensions—nitrogen and carbohydrates—that also reflect a suite of correlated nutritional components and whose relative value is nonlinear. For herbivores, nitrogen is a highly valued resource and our diets were designed so nitrogen concentrations were within the natural range of surrounding foliage (Bedoya-Perez et al. 2014b). Importantly, foods were designed to be complex—all co-varying across other nutritional dimensions, such as fiber, fat, vitamins and minerals—and nutritionally realistic for mammalian herbivores.

To make the dominating decoy present but inaccessible, we needed to provide some cue wallabies could associate with the foods to detect and identify them. In contrast to humans, whose dominant cue is often visual, wallabies use olfactory cues to locate, identify and select foods from afar (Bedoya-Perez et al. 2014a; Stutz et al. 2016; Finnerty et al. 2017). We therefore tested and confirmed that volatile organic compound (VOC) profiles differed among the foods, providing wallabies with cues they could associate with each food item (see material).

### Phase 1: preliminary binary preference trials

**Figure 3:**
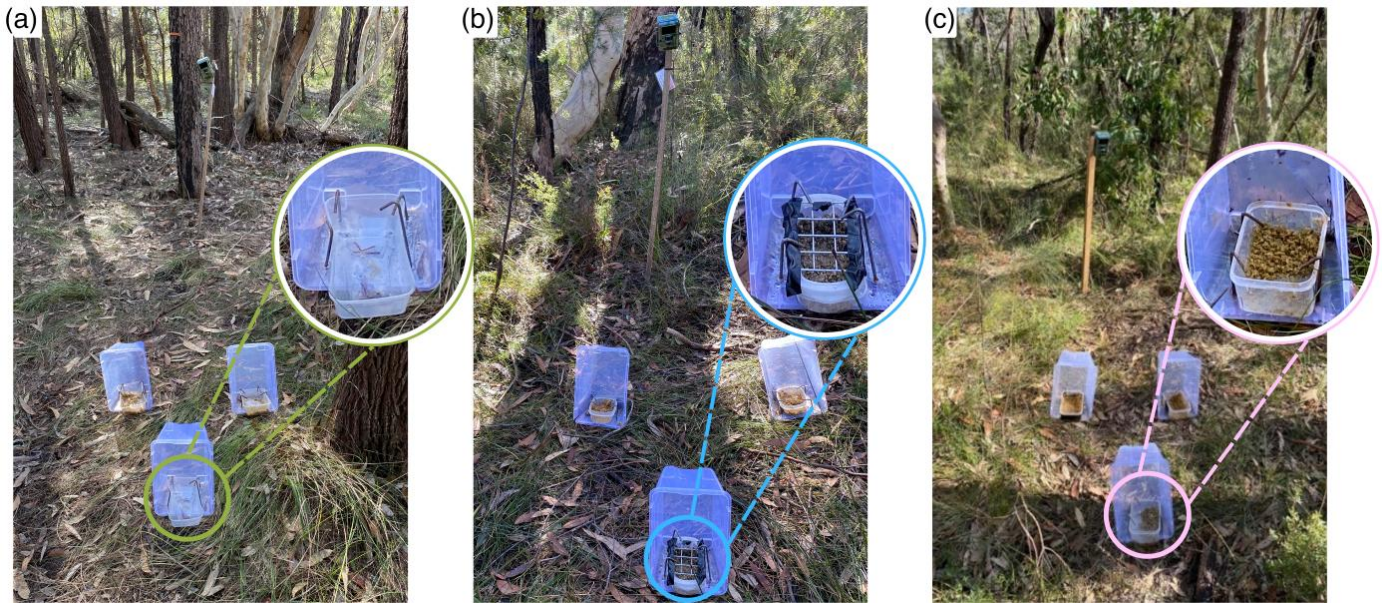
Experimental set up of treatments at a given plot. a) Binary treatment: available target and competitor foods are presented, as well as an empty container, for 3 days. b) Phantom treatment: available target and competitor foods are displayed for 3 days, as well as the dominating decoy food (the latter made unavailable for consumption on day 3 using a mesh lid). c) Trinary treatment: target, competitor and dominating decoy foods are all available for 3 days. Each food is presented in a container, with the 3 containers spaced 30 cm apart in a triangle. A camera at each plot recorded wallaby behaviors.

Within the study site, we set up 30 plots, each plot displaying 2 clear open food containers protected from rain, 30 cm apart (Fig. 3). Each plot had a motion triggered infrared camera (ScoutGuard SG560K or SG2060-K) set to 60 or 180 s videos (based on camera model) with instant retrigger, attached to a 1.2 m wooden stake and facing ∼ 45-degree downward to record wallaby visits and behaviors at the food containers. Plots were spaced ∼ 100 m apart to ensure independence of choices by wallabies among plots and to ensure we sampled a range of individual wallabies across plots.

We tested 6 pairs of food (binary treatments) consecutively, to select our target, competitor, and phantom decoy foods for *Phase* 2 (Table S1, Figure S1). For a given pair, at each plot (*n* = 30 replicates) we placed 1 food type (≈ volume, ∼10–20 g dry matter) per container.

In our first binary trial, wallabies took several days to display a preference, possibly as they learned about the nutritional quality and odor of these unfamiliar foods. We therefore ran each paired trial for 3 consecutive nights. Food was replaced each day and their location—left-hand side (LHS) or right-hand side (RHS)—was initially randomly allocated for each plot then switched each night to avoid a location bias.

We considered preference per day as the first food eaten and also quantified the percentage time spent eating either food in the first 60 s of feeding. A wallaby would typically consume the entirety of a food type in 60–80 s. Based on the results of these 6 trials (Figure S2), we selected target, competitor, and dominating decoy foods that lay in nutritional space with Dominating Decoy > Target > Competitor for nitrogen; and Competitor > Dominating Decoy = Target for carbohydrates (Fig. 1). These 3 foods were used in the main phantom decoy experiment—*Phase 2.*

### Phase 2: phantom decoy experiment

We used 45 plots, ∼100 m apart (30 in the same location as *Phase 1* and 15 new), with 3 containers and a camera at each plot as in *Phase 1*. We tested 3 treatments—Binary, Phantom, and Trinary (described in detail below)—15 replicates (plots) per treatment, per period, across 3 periods. Treatment was randomly allocated to plots in the first period, then via a cross-over design to balance treatments across time and space and account for potential carryover effects (Ratkowsky et al. 1993). Within each plot, the target and competitor were allocated to containers and, as in *Phase 1*, position of the 2 foods was switched each day within a given period. The dominating decoy food, or empty container, was placed 30 cm from both containers forming a triangle (Fig. 3).

As in *Phase 1*, we ran each experimental period for at least 3 consecutive days (4 days if a plot was unvisited on Day 3 to ensure we had a visit after the learning period—a “valid” treatment day). On Days 1 and 2 of each period, in all treatments, all foods were available for wallabies to consume to ensure the animals had the opportunity to learn the nutritional properties and match the odors to the specific foods on offer. Whether a food was available on Day 3 of a period depended on treatment.

#### Binary

Two available foods—target and competitor—in containers as in *Phase 1*, available for the entire period (Days 1–3). We also deployed an empty container as a procedural control for the absent dominating decoy food.

#### Phantom

Two available foods—target and competitor—in containers as in *Phase 1*, available for the entire period (Days 1–3). On Days 1–2, the dominating decoy food was available in a third container to ensure the wallabies were familiar with it. On Day 3, we made the dominating decoy food unavailable—a phantom decoy—placing a wire mesh lid on the container so wallabies could smell and see but not eat it.

#### Trinary

Two available foods—target and competitor—in containers as in *Phase 1*, and available dominating decoy food in a third container, all available for the entire period (Days 1–3). We included this treatment to account for any effect of changing 1 food (dominating decoy) from available to unavailable on Day 3 in the Phantom treatment.

For each period, we used data from Day 3 (the first valid treatment day ie Day 3 or 4) to quantify food choice and behaviors in response to treatments.

### Phase 3: follow-up binary preference trials

We next ran 2 follow-up binary preference trials, repeating: (i) dominating decoy versus target food, and (ii) dominating decoy versus competitor food. We did this because results from *Phase 2* Trinary treatment showed the available dominating decoy was chosen the least, despite it being the highest in nutritional value and significantly preferred to both the target and competitor foods in *Phase 1* binary trials. As such, we needed to check if binary preference between these foods had changed since *Phase 1*. A secondary aim was to confirm that food choice was consistent irrespective of the perceived amount of food offered. For each preference trial we therefore incorporated a treatment with comparing 2 extremes: *Volume* (paired foods matched for volume) and *Mass* (paired foods matched for dry mass), with 22 replicates per level, randomly allocated across a total of 44 plots. Each trial ran for 3 consecutive nights, and food location was determined as in *Phase 1*.

### Quantifying wallaby behavioral responses

In *Phase 1* and *Phase 3* binary preference trials, we quantified and compared wallaby behaviors across all nights. In *Phase 2*, we only quantified wallaby behaviors on Day 3 of each period. On a given night, we analysed only the first visit where a wallaby ate any food, as later visits may have been confounded by differing starting volumes of foods or by any animal odors.

Using Behavioral Observation Research Interactive Software (BORIS [Friard and Gamba 2016]), we quantified 2 variables, (i) preference as food first consumed (instant choice) and (ii) short-term preference as the food consumed the most (%) within the first 60 s of feeding. However, in 99% of visits the instant choice was the only food consumed across the short-term. Results of (ii) were therefore effectively the same as (i) and a more detailed measure of preference (such as % eaten of each food) was no longer necessary. We did not quantify long term preference over the entire visit, as wallabies usually finished the first food they chose, meaning consumption of the alternate food no longer fit within a binary choice framework. We also recorded other behaviors—including time spent overtly sniffing a food (distinctly pointing nose at food or sampling air), if a wallaby switched foods without finishing the first (yes/no), and if the food a wallaby first visited was the food eaten first (yes/no)—in case treatment influenced any exploratory behaviors, as found in Orlando et al. (2023). However, wallabies overtly sniffed food in < 10% of visits, a wallaby switched foods within the first minute in only 1 visit (1%), and a wallaby ate the first food they walked to in 88% of visits with little differences between treatments. We therefore did not consider these variables further.

### Statistical analyses for phase 1 and phase 3 binary preference trials

All statistical analyses were carried out in RStudio (R Core Team 2025). For *Phase 1*, we analysed each binary preference trial based on instant choice. For a given trial, we selected 1 of the 2 foods and quantified the response variable as whether it was eaten first (1 = yes, 0 = no). We ran a generalized linear mixed model (glmm) with a logit link function and binominal distribution using the glmer package lme4 and anova function (package: car) to test significance of the fixed effects. *Day* was included as a fixed factor and *plot* a random factor. Preferences were considered significant if 95% confidence intervals (CI) did not overlap 0.5. For *Phase 3*, first choice was analysed as above, including treatment (matched for Volume or Mass) as a fixed explanatory variable. Results were visualized with ggplot2 package, back-transformed into probability of choosing 1 food over the other.

### Statistical analyses for phase 2 phantom decoy experiment

#### Choice at group level

We first analysed the data at the group level comparing instant choice across Binary, Phantom, and Trinary treatments. We ran a glmm with a logit link function and binominal distribution using the lme4 package to quantify if food first chosen (target or competitor) was associated with treatment (Binary, Phantom, Trinary). Our response variable was *food first chosen* (1 = Target, 0 = Competitor) with *treatment* as the fixed explanatory variable and *plot* as a random factor. As the dominating decoy food was not available in Binary and Phantom treatments, we excluded samples where the dominating decoy was chosen first (n = 8) from the Trinary. While this made the data unbalanced, our main interest here was the comparison between Binary and Phantom treatments.

#### Choice at individual level

A potential problem with a group-level approach to the analysis is cross-level bias where overall group responses can mask individual changes (Edwards and Pratt 2009; Sasaki and Pratt 2011). To overcome this problem, we also analysed the data at the individual plot level (hereafter referred to as individual level) to test whether wallaby responses differed between (i) Binary versus Phantom, and (ii) Binary versus Trinary treatments. We considered each plot to be a valid approximation for this individual level analysis for several reasons. First, despite some overlap in home ranges, these wallabies are solitary, so visits are independent among wallabies. Second, we spread the plots out so no more than 1–2 plots were likely to fall within any individual's home range (Kaufmann 1974; Troy and Coulson 1993). Third, throughout our preference trials we found a consistent 2-day learning period for a given food at a given plot. This finding strongly indicates that the same individual, or small group of individuals, were visiting a given plot.

For each paired treatment comparison—(i) Binary versus Phantom, and (ii) Binary versus Trinary—we used the lme4 package in R and fitted a glm with a logit link function and binominal distribution. Our response variable was *switch in choice between treatments* (1 = yes, 0 = no) with *food eaten in Binary treatment* (target or competitor) as the fixed explanatory variable. In (i) Binary versus Phantom at a given plot, for example, if the target food was chosen in the Binary treatment, but the competitor was chosen in the Phantom treatment, then *switch in choice* = yes and *food eaten in Binary* = target for that plot. In (ii) Binary versus Trinary at a given plot, for example, if the target food was chosen in the Binary treatment, but the phantom decoy was chosen in the Phantom treatment, then *switch in choice* = yes and *food eaten in Binary* = target for that plot (as summarized in Table S2). We ran analysis of deviance on the model outputs using the car package to test significance of treatment on switching food choice. A switching effect was significant when the parameter estimates for the modeled log of the odds ratio was significantly different from zero. Results were visualized with the visreg package, back-transformed into probability of switching. As the experiment was run as a cross-over design, with treatment allocation balanced across period and space, with no treatment consistently preceding another treatment across plots, we avoided confounding period with any directional change in response between 1 treatment and its comparison pair.

## Results

### Preliminary binary preference trials

**Figure 4:**
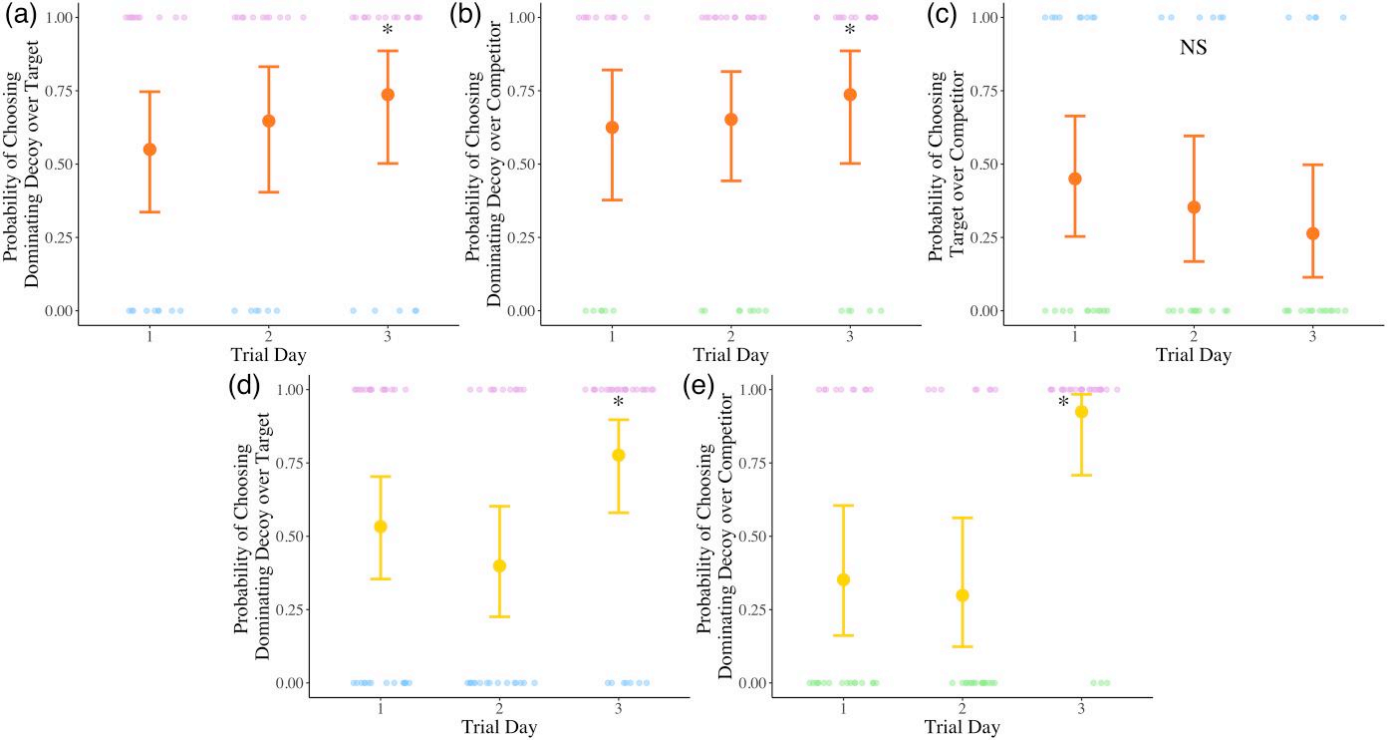
Probability of wallabies choosing 1 food item over another in binary preference trials over 3 trial days. a) Dominating decoy vs target in Phase 1, b) dominating decoy vs competitor in Phase 1, c) target vs competitor in Phase 1, d) dominating decoy vs target in Phase 3, e) dominating decoy vs competitor in Phase 3. Phase 1 and Phase 3 results are presented together to highlight there is no change in preference and food ranking (DD > T/DD > C) over time. Dotsdepict the food chosen per individual plot, points are the medians, and error bars represent 95% CIs. Asterix indicates significant difference (CI doesn’t bound 0.5), hence significant preference.

Here, we focus on preference trials between foods we selected for the target, competitor, and dominating decoy foods in *Phase 2* (results for 3 other preference trials did not yield appropriate foods for *Phase 2*; see supplementary information). In the binary preference trial comparing choice between the dominating decoy and target, wallabies were significantly more likely to choose the dominating decoy food (on Day 3, 95% CI: 0.502–0.886, Fig. 4a). Similarly, in the binary preference trial comparing choice between the dominating decoy and the competitor, wallabies showed a significant preference for the dominating decoy (on Day 3, 95% CI: 0.502–0.886 (by coincidence, exactly the same as above), Fig. 4b). In the binary preference trial comparing choice between the target and competitor, across all trial days wallabies displayed no significant preference for either option (on Day 3, 95% CI: 0.394–0.799, Fig. 4c). These binary preference trials confirmed that actual food preferences of the wallabies were consistent with the relative ranking of options described in conceptual models (ie, DD > T ≈ C).

### Phase 2: phantom decoy experiment

#### Choice at group level

**Figure 5:**
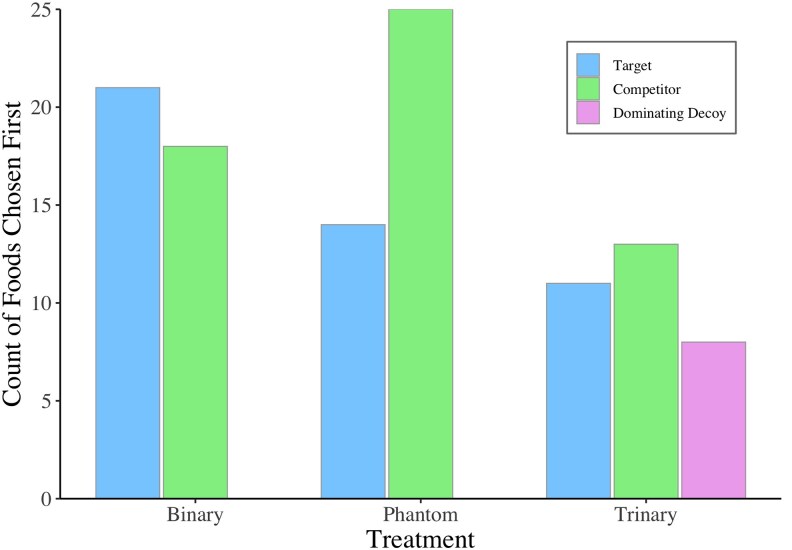
Count of the first food eaten—either target, competitor, or dominating decoy—on day 3 in binary, phantom and trinary treatments across all periods in phase 2.

**Figure 6:**
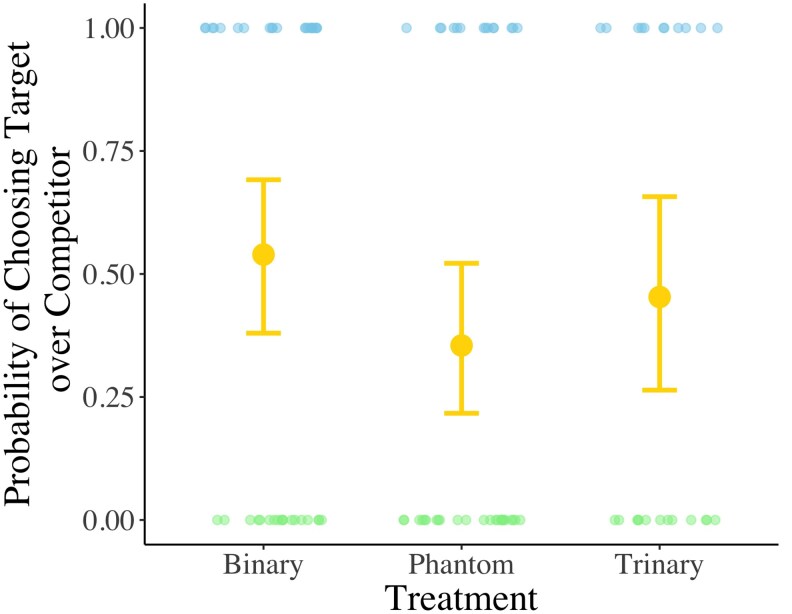
Probability of wallabies choosing target food over competitor on day 3 in binary, phantom and trinary treatments across all periods in phase 2.

Across the 3 periods, there were 110 independent swamp wallaby visits, with 39, 39, and 32 visits to Binary, Phantom, and Trinary treatments respectively. Using the Binary treatment as the baseline, in the Phantom treatment there was a net increase in choice for the competitor. Unexpectedly, in the Trinary treatment, the available dominating decoy food was chosen least (Fig. 5). However, there was no significant association in food first chosen (target or competitor) and treatment (Binary, Phantom, and Trinary) (χ^2^ = 2.5162, df = 2, *P* = 0.284, Fig. 6).

#### Choice at individual level

**Figure 7:**
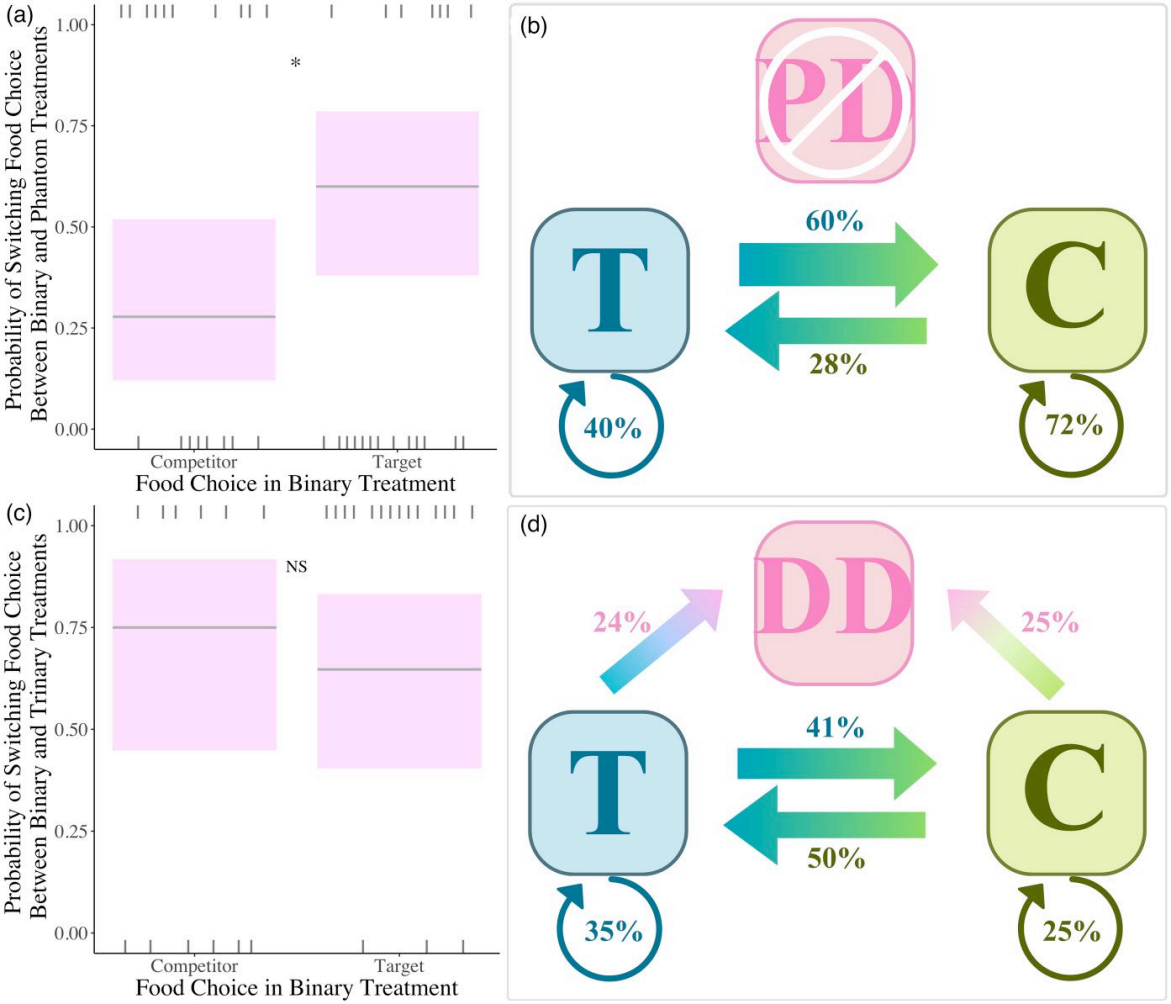
Probability of wallabies switching food choice at the individual level between a) Binary and phantom treatments and c) Binary and trinary treatments in phase 2. Dashes show the number of choices for each food, the gray line depicts the median, and the shaded regions represent the CI (95%). Counts of wallaby food choice for b) target or competitor between Binary and Phantom treatments or, d) target, competitor, or dominating decoy between Binary and Trinary treatments at an individual level. Straight arrows depict individuals switching food choice between treatments and circular arrows depict individuals with consistent choices between treatments.

In the pairwise comparison of individual choices between Binary and Phantom treatments, there was a significant effect of the food chosen in the Binary and the probability of switching food choice from Binary to Phantom treatments (χ^2^ = 4.067, df = 1, *P* = 0.044, Fig. 7a). Wallabies that chose the target in the Binary treatment were more likely to switch choice to the competitor in the Phantom treatment. In contrast, wallabies that chose the competitor in the Binary treatment were more likely to continue to choose the competitor in the Phantom treatment (Fig. 7b). In the pairwise comparison of individual choices between Binary and Trinary treatments, there was no evidence that the food chosen in the Binary affected the probability of switching food choice from Binary to Trinary treatments (χ^2^ = 0.353, df = 1, *P* = 0.552, Fig. 7c). Furthermore, and contrary to our expectation based on binary preference trials in Phase 1, wallabies switched their choice from either target or competitor foods to the available dominating decoy food in only ∼25% of cases (Fig. 7d).

### Phase 3: follow-up binary preference trials

In *Phase* 3 binary preference trials, wallabies showed a significant preference for the dominating decoy food over the target (on Day 3, 95% CI: 0.582–0.897, Fig. 4d) and competitor (on Day 3, 95% CI: 0.700–0.984, Fig. 4e) as they did in *Phase 1*. There was no effect of treatment (Volume/Mass) on choice between the dominating decoy and target (χ^2^ = 0.08, df =1 *P* = 0.77, Figure S3a) or the dominating decoy and competitor (χ^2^ = 3.11, df = 1, *P* = 0.07, Figure S3b).

## Discussion

By taking a new approach to test individual level decisions, we demonstrate that an unavailable phantom decoy significantly shifted choice by wallabies, with a stronger net shift away from the target to the competitor food. Unexpectedly, in the Trinary treatment individual choice changed again, with the most preferred food in binary tests (ie the available dominating decoy food) chosen the least. These individual results were reflected in patterns at the group level, but the group level changes were not significant, indicating cross-level bias. Our findings directly build on a previous study with wallabies (Orlando et al. 2023), showing phantom decoys not only influence information processing behavior by wild mammals before they make a choice, but also in what they choose, ie the decision outcome. Together, these findings extend our understanding of cognitive processes of animals under realistic scenarios. They also have important implications for how we describe, understand, and ultimately predict foraging decisions of free-ranging animals.

### Phantom decoy changes choice—a net reactance effect

Overall, the unavailable phantom decoy increased choice for the competitor, hence decreased choice for the target food. This pattern is consistent with a net reactance effect at the population level. From a cognitive perspective, the reactance effect is suggested to arise from “annoyance” or “attention”. In the first, an individual may become frustrated (annoyed) they cannot have what they want (the phantom decoy) so they “rebel” and choose the most different option—the competitor (Tversky 1972; Scarpi and Pizzi 2013). In the second, adding the phantom decoy induces a salience bias so the individual focuses on and attends to the most unique option (Tsetsos et al. 2012), once again the competitor (Spektor et al. 2019). Whether we could identify such psychological drivers underlying wild animal decisions, especially without anthropomorphizing, remains a challenge.

Two additional factors, relating to how the choice task is structured, may also cause shifts in choice, towards either the competitor or the target. These nonmutually exclusive factors are (i) attribute “concreteness” of the options, and (ii) relative distance in value of the decoy versus the target along their common dimension. When options have high attribute concreteness (ie their values are explicit, eg stated or numeric) (Pettibone and Wedell 2000; 2007) people have been shown to shift choice towards the target. But when options have low attribute concreteness (ie values/properties have to be inferred), people shift choice towards the competitor (Trueblood and Pettibone 2017; Spektor et al. 2019; Spektor et al. 2021). Our target and competitor foods likely had low concreteness as the 2 foods had similar (but still distinct) odor profiles (see material) and wallabies use odor to evaluate food quality (Stutz et al. 2016; Finnerty et al. 2017). Their choice shift towards the competitor therefore appears consistent with responses of people.

In terms of distance in value, a decoy “far” in value from the target (> 50% separation on the dominated attribute) can shift people's choice towards the competitor, while a “close” decoy can shift people's choice towards the target (Scarpi and Pizzi 2013; Liao et al. 2021). In this study, we classify the phantom decoy as “far” in value from the target. Food nitrogen was the dominated attribute, and the phantom decoy had 2.8 times as much as the target (Fig. 1). This difference is highly meaningful from a nutritional perspective for herbivores, since plant (food) nitrogen is often a limiting nutrient (Robbins 1993). The target food nitrogen content (0.8%) was designed to mimic common low quality foliage whereas the phantom decoy food nitrogen content (2.24%) mimicked high-quality young foliage (Loney et al. 2006), a highly valuable resource. Furthermore empirical evidence shows that wallabies harvest food with nitrogen matching the phantom decoy much more than food with nitrogen matching the target (Bedoya-Perez et al. 2014b). As the “far” phantom decoy shifted choice by wallabies towards the competitor, results are consistent with responses of people. Wild animals—not just people—may therefore be sensitive to the relative value of options.

In contrast to our findings and human studies, in captive animal studies phantom decoy effects have only resulted in a shift towards the target—in line with the similarity-substitution hypothesis. Examples include food choice tasks with captive capuchin monkeys (Marini et al. 2024), honeybees (Tan et al. 2015) and domestic cats (Scarpi 2011), and mate choice with captive túngara frogs (Lea and Ryan 2015). Until more phantom decoy studies are done with wild animals in complex environments, it is unclear whether this directional disparity is due to experimental simplicity (captive) versus complexity (wild), or other factors such as attribute concreteness and distance in value.

### Binary-preference influences the phantom decoy effect

The net shift towards the competitor (reactance effect) by wallabies in the presence of the phantom decoy arose from a mix of individual responses linked to their preferences in the Binary treatment. Specifically, those preferring the target over the competitor in the Binary treatment were more likely to shift than those who initially preferred the competitor. Individual shifts that depend on *binary-preference* are often not considered as a defining condition for switching choice in phantom decoy studies. But investigating the influence of *binary-preference* on decisions may advance our understanding of decoy effects and what influences or moderates them: *binary-preference* may be a crucial component.

### More options lead to suboptimal choice: due to cognitive overload?

In the Trinary treatment in *Phase 2*, wallabies chose the available dominating decoy food least often. We expected them to choose it most, given its high nutritional value and the fact wallabies strongly preferred it over the target and competitor foods in binary preference trials both before (in *Phase 1*) and after (in *Phase 3*) *Phase 2*. It is highly unlikely wallabies changed food preferences coincidentally in this middle 3-wk window of *Phase 2*. Instead, the change seems linked to the increase in choice set size, from 2 to 3. Such an increase does not seem much in absolute terms, but it is a 50% increase. In addition, wallabies had to choose in an already noisy, complex environment, processing and filtering information about the artificial foods and all other plants in the vicinity of the plots. Too many options may have exceeded the cognitive processing capacity of the wallabies, leading to cognitive overload, in turn leading to slow or suboptimal choices (Chernev et al. 2015). This explanation is consistent with studies on choice overload in people (Misuraca et al. 2024) and emerging research in nonhuman taxa (Tanner and Hemingway 2025) and most notably findings with bumblebees, in which an increase in flower choice, from 2 to 4, led to higher constancy and decision-latency—associated with foraging costs (Austin et al. 2019). Whether wallabies would respond similarly, using only real plants, remains to be seen.

### Decoys as a cue for making heuristic decisions

Taken together, our Phantom and Trinary results suggest a heuristic basis for decoy effects in free-ranging wallabies. Heuristics or “rules-of-thumb” are strategies individuals can employ that harness a subset of information to make fast, “good enough” decisions (Hutchinson and Gigerenzer 2005; Gigerenzer and Gaissmaier 2011). While wallabies made suboptimal choices in the Trinary treatment, in the Phantom treatment they may have harnessed the unavailable phantom decoy food as a point of reference to compare the available foods, thus eliciting a heuristic response (Tsetsos et al. 2012). Recent human studies have found that decoy effects diminish as choice set size increases (2 to 3 and beyond) (Stanley and Wedell 2024; Marini et al. 2025), perhaps because the decoy's role as a “point of reference” may become obfuscated with more comparisons (decoy vs option 1, decoy vs option 2…). Future research could explore decoy effects in complex choice scenarios with free-ranging animals to further reveal when and how decoys are harnessed for heuristic decision-making.

### At the crossroads of cognition and foraging

This study provides the first empirical evidence of phantom decoys influencing decision outcomes in free-ranging animals in natural environments. Such a phantom decoy effect appears to contradict the fundamental premise of foraging theory—that individuals will make optimal food choices using an absolute evaluation system (Marsh 2002; Hutchinson and Gigerenzer 2005). Since they cannot be contradictory, there must be a logical way to reconcile the 2. Incorporating the influence—and presumably adaptive benefits—of heuristic and cognitive shortcuts into foraging theory should do just that. Investigating how, when, and why foraging animals harness decoy information—and the role of heuristics in such decisions—will reveal cognitive mechanisms and shortcuts that influence foraging decisions. Importantly, it should also improve our understanding of foraging behavior by wild animals, improve traditional foraging models, and better reflect real-world foraging.

## Supplementary Material

arag059_Supplementary_Data

## Data Availability

Analyses reported in this article can be reproduced using the data available at https://doi.org/10.5061/dryad.2jm63xt3w Jarvis et al (2026).
