# Supplementary material for: A phantom decoy shifts wild forager decisions in a natural environment
Source: Behav Ecol. 2026 May 30;37(4):arag059. doi: 10.1093/beheco/arag059 (PMC13270240; doi:10.1093/beheco/arag059)
Supplement: arag059_Supplementary_Data [file arag059_supplementary_data.docx]

A Phantom Decoy Shifts Wild Forager Decisions in a Natural Environment: Supplementary Material

*Behavioral Ecology*

Table 1. Foods tested in binary preference trials and the percentages of nitrogen, sugar, and estimate fibre of each food item. Nitrogen was quantified using an elemental analyser (Elementar Vario Max CNS, Analysensystem GmbH, Hanau, Germany).

| *Diets* | Rabbit pellets (g) | Oaten Hay (g) | Sugar (%) | Nitrogen (%) | Fibre (%) |
| --- | --- | --- | --- | --- | --- |
| A | 50 | 20 | 30 | 1.23 | 12.5 |
| B | 90 | 0 | 10 | 2.06 | 4.5 |
| C | 55 | 0 | 45 | 1.26 | 2.8 |
| F | 98 | 1 | 1 | 2.24 | 5.4 |
| G | 40 | 59 | 1 | 1.17 | 31.5 |
| Target (K) | 20 | 79 | 1 | 0.8 | 40.5 |
| Competitor (L) | 5 | 90 | 5 | 0.51 | 45.3 |
| Dominating/Phantom Decoy (J) | 98 | 1 | 1 | 2.24 | 5.4 |


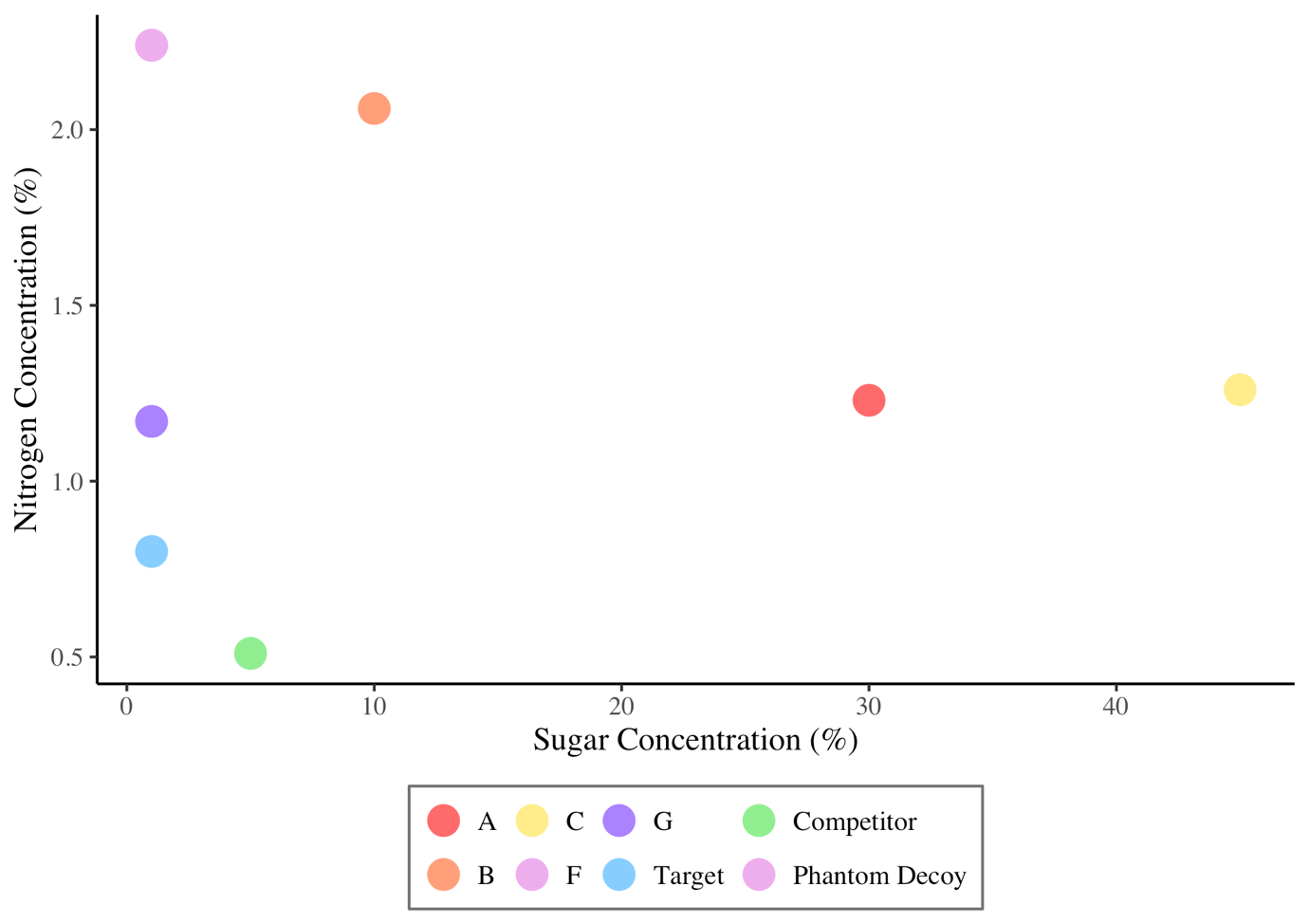


Figure 1. The location in 2-dimensional nutritional space, in terms of percentage nitrogen and sugar, for all foods (A-L) tested in binary preference trials

## Preference Trials: A vs B, B vs C, F vs G

In the binary preference trials comparing foods A vs B, B vs C, and F vs G wallabies displayed no significant preference for either option, represented by confidence intervals binding 0.5 (Figure 2).

b)

a)


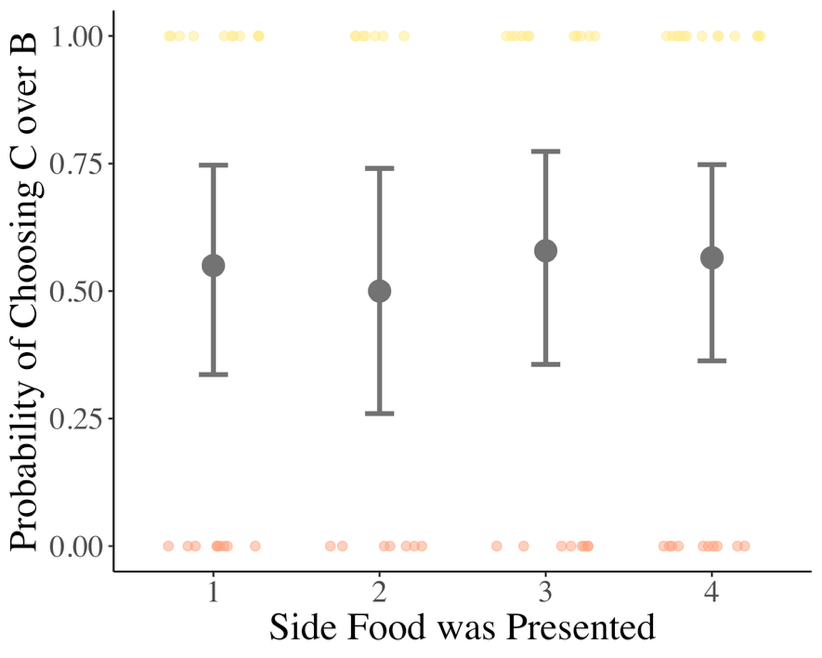

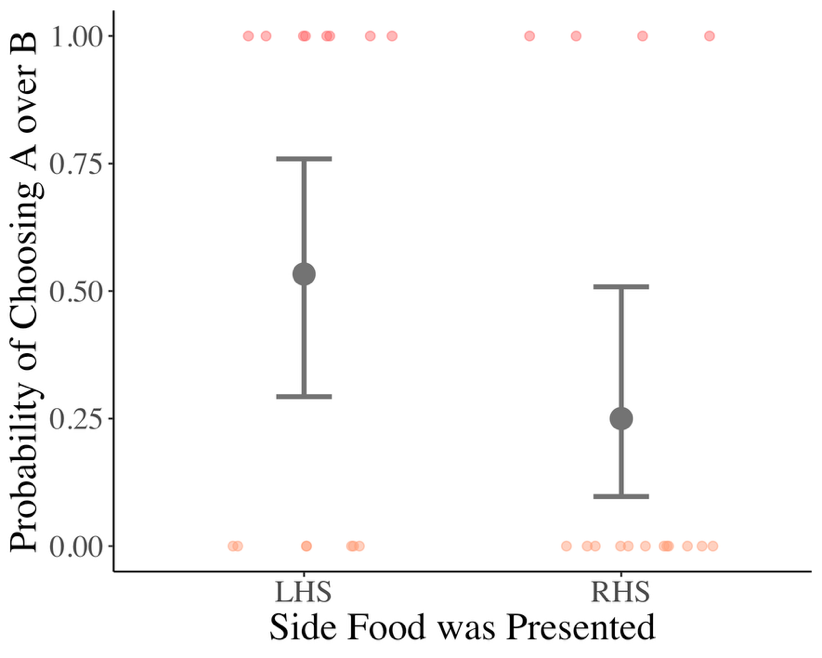


c)


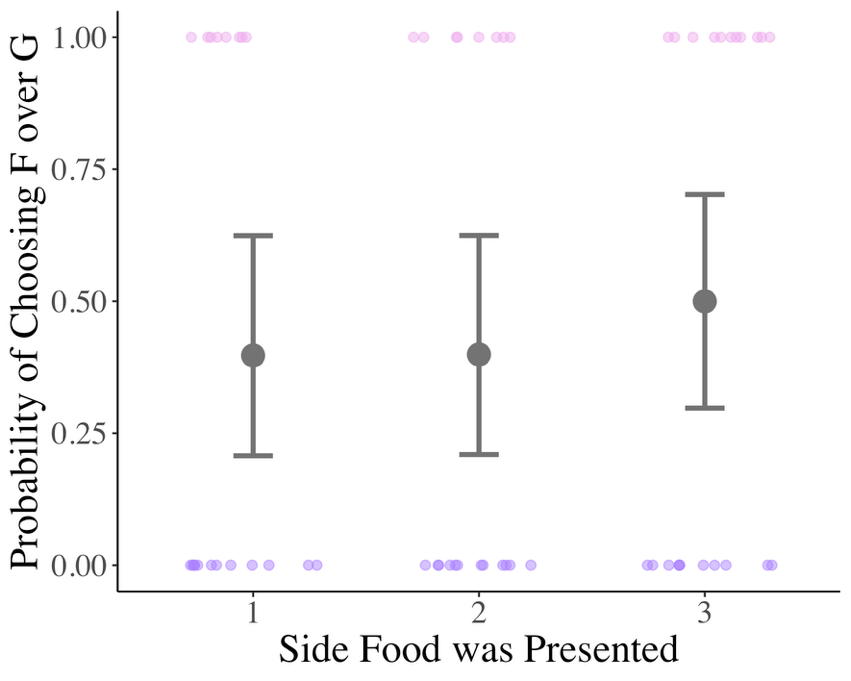


Figure 2: a) probability of swamp wallabies choosing food A over food B at first choice based on feeder, b) probability of swamp wallabies choosing food C over food B at first choice across trial days, c) Probability of swamp wallabies choosing food F over food G at first choice across trial days. Dots show number of choices for each food, 95% confidence intervals.

## Mass vs Volume Preference Trials

In Phase 2, the three foods (target, competitor, and dominating decoy) were matched for volume. As two major ingredients that we used - rabbit pellets and ground hay —differed markedly in density (dry mass per volume [grams/ml]), when we varied the amount of these ingredients to make the three foods, the density of the resulting foods also differed.

While conducting binary preference trials in *Phase 3* to check whether binary preference had changed between phases, we took the opportunity to confirm that preference for the dominating decoy was the same, irrespective of whether we matched it against the other food option by volume or dry mass. For each preference trial we therefore incorporated a treatment with two levels: *Volume* (paired foods matched for volume) and *Mass* (paired foods matched for dry mass). There was no effect of treatment (Volume/Mass) on choice between the phantom decoy and target or the phantom and competitor (Figure 3).


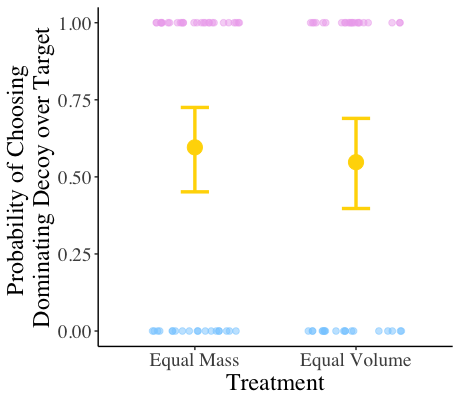

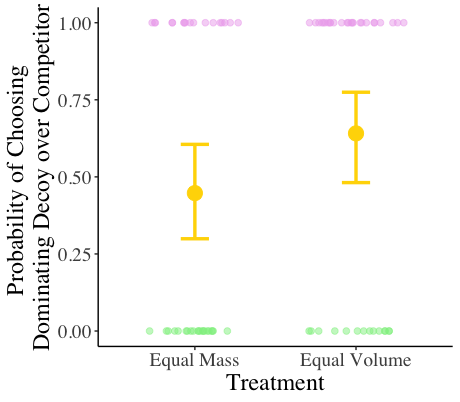


a)

b)

Figure 3: a) Probability of swamp wallabies choosing food J over food K at first choice based on treatment, b) Probability of swamp wallabies choosing food J over food L at first choice based on treatment. Dots show number of choices for each food, 95% confidence intervals.

Table 2: Summary of response and explanatory variables in accordance with food choice across treatments used in the model *switch in choice between treatments* (1 [yes], 0 [no]) ~ *food eaten in Binary treatment* + (1|plot).

| **Food Choice** | | | **Response Variable** | **Explanatory Variable** |
| --- | --- | --- | --- | --- |
| ***Binary*** | ***Phantom*** | ***Trinary*** | ***switch in choice*** | ***food eaten in Binary*** |
| Target | Target |  | No (0) | Target |
| Target | Competitor |  | Yes (1) | Target |
| Competitor | Target |  | No (0) | Competitor |
| Competitor | Competitor |  | Yes (1) | Competitor |
| Target |  | Target | No (0) | Target |
| Target |  | Competitor | Yes (1) | Target |
| Target |  | Dominating Decoy | Yes (1) | Target |
| Competitor |  | Target | Yes (1) | Competitor |
| Competitor |  | Competitor | No (0) | Competitor |
| Competitor |  | Dominating Decoy | Yes (1) | Competitor |

### Volatile Organic Compound Analyses

#### Volatile Organic Compound sampling

Each food was placed in a regular (25 x 38 cm) GLAD® oven bag for 10 minutes to allow the odour to fill the headspace. Two blanks were run for each set (for every 4 samples), consisting of empty plastic containers in oven bags. Headspace air for the samples and blanks were then sampled for volatile organic compounds (VOCs) for 15 minutes at 60 mL min^-1^ (Spektrex PAS-500 Micro Air Sampler pump) through thermal desorption (TD) tubes (200 mg Tenax TA; Markes International Ltd). Six replicates of each food were run, and TD tubes were stored at 4°C until processing (approximately five days after sampling).

#### Volatile Organic Compound analysis

Thermal desorption (TD) tubes were processed as described in (1) with some modification. TD tubes were desorbed at 300 °C for 6 min using an automated thermal desorption unit (Ultra-2 and Unity-2; Markes International, Bridgend, UK.) and concentrated at -30 °C on a Tenax TA cold trap.  Following flash heating of the cold trap to 300 °C, injection to the GC (Agilent 7890A; Agilent Technologies Pty Ltd; Mulgrave, VIC, Australia) was made via a transfer line held at 150 °C. A 60 m × 0.32 mm, 1 μm film thickness DB-1 capillary column (Agilent) was fitted to the GC and run splitless at a flow rate of 2.3 mL min^− 1^. The GC oven was heated to 35 °C for 5 min, followed by 4 °C min^− 1^ to 160 °C, then 20 °C min^− 1^ to 300 °C and held for 5 min. A mass-selective detector (Model 5975 C; Agilent), coupled to the GC, was set to a scan range of 35–300 amu.

Common contaminating ions (73, 84, 147, 149, 207, 221 and 281 m/z) were removed from the chromatography using the denoising function in OpenChrom Lablicate Edition (version 1.1.0, Lablicate GmbH, Germany). Further pre-processing was performed using the metaMS package (2) through the Galaxy Project platform (The Galaxy Community, 2022) Workflow4Metabolomics (3).

After background subtraction, there was a total of 83 identified VOCs, 35 for dominating decoy, 31 for target, and 26 for competitor foods L.

An ANOSIM on the 4th root transformation of the total ion counts reported a significant difference between the emitted VOCs and foods (R = 0.366, p = 0.001). Pairwise comparisons show that VOCs were significantly different between phantom decoy and target foods (R = 0.587, p = 0.002) and phantom decoy and competitor foods (R = 0.57, p = 0.002), but no significant difference was found between the target and competitor emissions (R = -0.072, p = 0.76). nMDS analyses visually represent these patterns, showing that the target and competitor samples are interspersed among each other and the phantom decoy samples are distinctly clustered together (Figure 4).


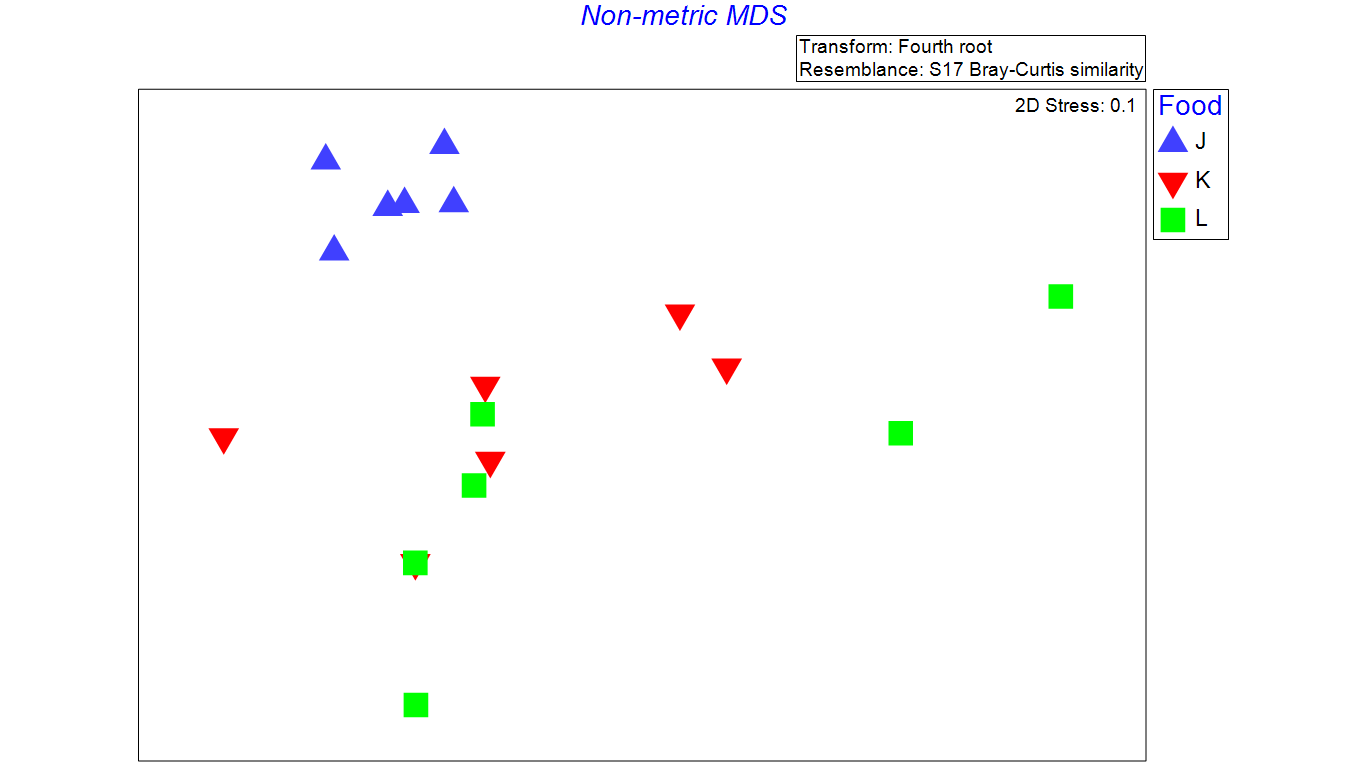


Figure 4: nDMS plot using Bray-Curtis similarities of the odour profiles of dominating decoy (J), target (K), and competitor (L) foods

# References

1. Lawson CA, Seymour JR, Possell M, Suggett DJ, Raina J-B. The Volatilomes of Symbiodiniaceae-Associated Bacteria Are Influenced by Chemicals Derived From Their Algal Partner. Frontiers in Marine Science. 2020;7.

2. Wehrens R, Weingart G, Mattivi F. metaMS: An open-source pipeline for GC-MS-based untargeted metabolomics. Journal of Chromatography B-Analytical Technologies in the Biomedical and Life Sciences. 2014;966:109-16.

3. Giacomoni F, Le Corguillé G, Monsoor M, Landi M, Pericard P, Pétéra M, et al. Workflow4Metabolomics: a collaborative research infrastructure for computational metabolomics. Bioinformatics. 2015;31(9):1493-5.
